# Editorial: Driver Behavior and Performance in an Age of Increasingly Instrumented Vehicles

**DOI:** 10.3389/fpsyg.2021.715239

**Published:** 2021-07-28

**Authors:** Oren Musicant, Haneen Farah, David Shinar, Christian Collet

**Affiliations:** ^1^Department of Industrial Engineering & Management, Ariel University, Ariel, Israel; ^2^Department of Transport & Planning, Delft University of Technology, Delft, Netherlands; ^3^Department of Industrial Engineering & Management, Ben-Gurion University of the Negev, Beersheba, Israel; ^4^Inter-University Laboratory of Human Movement Biology - Mental Processes, Cerebral Plasticity and Motor Performance Team (MP3), Université Claude Bernard Lyon 1, Lyon, France

**Keywords:** driver behavior, driver performance, advanced driver assistance system, older (elderly) drivers, autonomuos vehicles

Driver behavior and performance have been studied extensively in the last decades. Researchers have developed theories and models of driver situational awareness, driver decision-making and errors, information processing, and mental workload. Meanwhile, other scholars have focused on creating and optimizing in-vehicle technology-based interventions to increase safety. The purpose of this special issue is to bring together a collection of empirical and theoretical work focusing on understanding Driver Behavior and Performance in an Age of Increasingly Instrumented Vehicles. Several themes characterize this special issue:

## Advanced Driver Assistance Systems (ADAS)

ADAS offer timely advice and feedback and can even actively take (or yield) control of the vehicle. This particular theme includes several applications of future ADAS. Ahmed et al. studied the usefulness of a connected ADAS that presents information from a control center. A group of professional drivers experienced the connected ADAS in a driving simulator. They indicated that the messages from the ADAS were most helpful when visibility was poor. In addition, the most valuable warnings were forward-collision (with other connected vehicles) and rerouting. Such findings are helpful for designers of ADAS that can receive information from control centers. In an interesting naturalistic study, Davis et al. discovered different behavioral patterns for elderly drivers: with Alzheimer disease (group 1), without Alzheimer disease (group 2), and with early signs of Alzheimer disease (group 3). The early Alzheimer disease group (#3) had fewer speeding and g-based driving events per driving distance traveled than the other groups. This result indicates sufficient orientation to self-regulate risk-taking while driving. Taking a broader perspective, this study provides an example of how ADAS can help detect (and perhaps take preventive measures) in situations related to driver health, e.g., when transferring from early signs to more advanced phases of the disease. Therefore, this theme is linked to the next theme in our Research Topic—the driver state monitoring theme (described below). ADAS technology may also have adverse outcomes. ADAS may cause deterioration of driving skills, encourage the diversion of attention from the driving task to other stimuli, and impair risk perception. Cohen-Lazry and Borowsky studied a novel multi-touch interface for an in-vehicle infotainment system and compared it with a functionally similar control interface. Participants using the multi-touch interface needed less time to complete secondary tasks, were quicker at identifying potential hazards, and reported lower subjective workload.

## Driver State Monitoring

The second theme that characterizes several studies in this special issue is driver state monitoring. Current in-vehicle sensors can detect functional indices such as head position, eyelid, gaze, respiration rate, heart rate, and skin conductance to infer fatigue, mental workload, distraction, and risk perception. A significant portion of the studies in this special issue focused on using objective physiological indices to estimate mental workload and stress. For example, even in a simple common task, as searching for a parking space, Ponnambalam and Donmez found marginally significant changes in skin conductance and heart rate, indicating an increased mental workload. In conditional automation, drivers may engage in secondary tasks that increase the mental workload and impair driving performance. To continuously monitor the driver's mental workload, Meteier et al. recommend a combination of physiological indices (based on skin conductance, electrocardiogram, and respiration), the length of the time window for data processing, and machine learning models to identify elevated states of mental workload. Meteier et al. focused on measuring mental workload during a continuous performance of secondary tasks. But what about more discrete events without secondary tasks? Sahar et al. found that even everyday braking events trigger physiological responses of heart rate and heart rate variability. Moreover, they tested a novel intuitive, instantaneous, and unintrusive measure of stress—the steering wheel grip force (i.e., the force applied on the steering wheel in a non-steering-related task). They found that the steering wheel grip force correlated with braking intensity (a performance index), heart rate, and heart rate variability (stress indices), demonstrating its validity as a measure of stress. Another exciting research direction within the driver state monitoring theme is searching for drivers' hazard perception indicators. One option is to use biomarkers for hazard anticipations. In their study, Chirles et al. found that experienced drivers (compared to learners) had greater electrodermal responses to hazards in a hazard perception test before the hazard manifested itself.

## AGE

Older drivers are known to make adjustments and self-regulation to accommodate cognitive, sensory, and motor capabilities changes. Such adjustments involve, for example, reducing long-distance drives. Here too, ADAS may be helpful. Shichrur et al. found that older drivers that used ADAS that provide collision warnings almost doubled their travel distance compared to the period before using the ADAS. It is possible that older drivers feel that the technology helps them compensate for reduced skills. Ironically, older drivers also struggle the most to adapt to ADAS technology. For example, according to Cooper et al., older drivers (compared to younger drivers) experienced increased workload when interacting with in-vehicle information systems: Older drivers were slower to respond to visual task demands and required more time to complete tasks such as entering a navigation destination, texting, calling, and dialing, and tuning the radio. These results remain consistent across three Human Machine Interfaces (HMIs) (two visual-motor and one vocal-based interaction). Although not obvious, human factors still play an essential role even in a fully autonomous vehicle. Stephenson et al. looked at the physiological responses of older passengers in an autonomous vehicle who faced expected and unexpected stops. After the unexpected stops, skin conductance sensors indicated increased passenger stress. This result suggests a need for interventions to reduce stress from unexpected events. Taking a broader perspective, the use of physiological sensors can serve to monitor passengers' stress as well as driver stress.

## Conclusion

In the coming years, the human factor will continue to have an essential role in driving. The driver behavior and performance special issue includes studies examining drivers' behavior and state considering a range of autonomous levels. Emerging topics include novel methods in sensing and inferring driver states, novel human-machine interfaces, novel ADAS capabilities, and increasing interest in the elderly population that can benefit the most from ADAS yet have the greatest difficulty in adopting it.

## Author Contributions

All authors listed have made a substantial, direct and intellectual contribution to the work, and approved it for publication.

## Conflict of Interest

The authors declare that the research was conducted in the absence of any commercial or financial relationships that could be construed as a potential conflict of interest.

## Publisher's Note

All claims expressed in this article are solely those of the authors and do not necessarily represent those of their affiliated organizations, or those of the publisher, the editors and the reviewers. Any product that may be evaluated in this article, or claim that may be made by its manufacturer, is not guaranteed or endorsed by the publisher.

